# Epstein-Barr virus-encoded miR-BART6-3p inhibits cancer cell metastasis and invasion by targeting long non-coding RNA *LOC553103*

**DOI:** 10.1038/cddis.2016.253

**Published:** 2016-09-01

**Authors:** Baoyu He, Weiming Li, Yingfen Wu, Fang Wei, Zhaojian Gong, Hao Bo, Yumin Wang, Xiayu Li, Bo Xiang, Can Guo, Qianjin Liao, Pan Chen, Xuyu Zu, Ming Zhou, Jian Ma, Xiaoling Li, Yong Li, Guiyuan Li, Wei Xiong, Zhaoyang Zeng

**Affiliations:** 1 The Key Laboratory of Carcinogenesis of the Chinese Ministry of Health, Xiangya Hospital, Central South University, Changsha, Hunan, China; 2 The Key Laboratory of Carcinogenesis and Cancer Invasion of the Chinese Ministry of Education, Cancer Research Institute, Central South University, Changsha, Hunan, China; 3 Hunan Key Laboratory of Nonresolving Inflammation and Cancer, Disease Genome Research Center, The Third Xiangya Hospital, Central South University, Changsha, Hunan, China; 4 Hunan Key Laboratory of Translational Radiation Oncology, Hunan Cancer Hospital and The Affiliated Cancer Hospital of Xiangya School of Medicine, Central South University, Changsha, Hunan, China; 5 Clinical Research Institution, The First Affiliated Hospital, University of South China, Hengyang, Hunan, China; 6 Department of Cancer Biology, Lerner Research Institute, Cleveland Clinic, Cleveland, OH, USA

## Abstract

Epstein-Barr virus (EBV) infection is causatively related to a variety of human cancers, including nasopharyngeal carcinoma (NPC) and gastric cancer (GC). EBV encodes 44 mature miRNAs, a number of which have been proven to promote carcinogenesis by targeting host genes or self-viral genes. However, in this study, we found that an EBV-encoded microRNA, termed EBV-miR-BART6-3p, inhibited EBV-associated cancer cell migration and invasion including NPC and GC by reversing the epithelial–mesenchymal transition (EMT) process. Using microarray analysis, we identified and validated that a novel long non-coding RNA (lncRNA) *LOC553103* was downregulated by EBV-miR-BART6-3p, and *LOC553103* knockdown by specific siRNAs phenocopied the effect of EBV-miR-BART6-3p, while *LOC553103* overexpression promoted cancer cell migration and invasion to facilitate EMT. In conclusion, we determined that EBV-miR-BART6-3p, a microRNA encoded by oncogenic EBV, inhibited EBV-associated cancer cell migration and invasion by targeting and downregulating a novel lncRNA *LOC553103*. Thus, our study presents an unreported mechanism underlying EBV infection in EBV-associated cancer carcinogenesis, and provides a potential novel diagnosis and treatment biomarker for NPC and other EBV-related cancers.

Epstein-Barr virus (EBV) is a gamma herpesvirus, which infects more than 90% of the world's adult population.^1^ Its latent infection is associated with a number of malignancies, including multiple types of Burkitt's lymphoma, Hodgkin's disease, nasal natural killer/T-cell lymphoma, nasopharyngeal carcinoma (NPC) and gastric carcinoma (GC).^2, 3, 4, 5, 6^ It produces several viral oncoproteins, including six EBV-encoded nuclear antigen proteins (EBNA-1, EBNA-2, EBNA-3A, -3B, -3C and EBNA-LP) and three latent membrane proteins (LMP1, LMP2A and LMP2B), which are expressed during different latency programs.^7^ Latent membrane protein 1 (LMP1), EBV nuclear antigen 2 (EBNA2) and EBNA3 genes are well-known EBV oncogenes that prevent cells from death and facilitate cell division.^8^ EBV also expresses two transcription factors during the immediate-early stage of the lytic cycle, replication and transcription activator (Rta) and BZLF1 transcription activator (Zta). Rta and Zta expression inhibits cell proliferation and results in cell cycle arrest through induction of cyclin-dependent kinase inhibitors.^9, 10, 11, 12^

Recently, it has become apparent that EBV encodes for a large number of microRNAs (miRNAs), including BART cluster and BHRF cluster.^13, 14^ The largest set of miRNAs is the *Bam*HI A rightward transcript (BART) miRNAs, which is composed of 40 miRNAs expressed in all forms of EBV latency.^13^ They were reported to promote viral latency or cancer development by targeting both viral and cellular genes.^15, 16, 17, 18, 19, 20, 21, 22^

We previously established a comprehensive EBV-miRNA profiles in NPC and also found that 40 EBV miRNAs from the BART transcript were highly expressed in NPC.^23^ In this study, we investigated the role of EBV-miR-BART6-3p in NPC and GC, two EBV-associated epithelial cancers. Unexpectedly, unlike the majority of the BART cluster miRNAs, we found that EBV-miR-BART6-3p reversed the epithelial–mesenchymal transition (EMT) phenotype, and inhibited cancer cell migration and invasion. We hypothesized that EBV-miRNA-BART6-3p might participate in EMT phenotype to inhibit cell migration and invasion by targeting and downregulating a novel lncRNA *LOC553103.*

## Results

### EBV-miR-BART6-3p suppressed tumor cell migration and invasion

**Figure 1:**
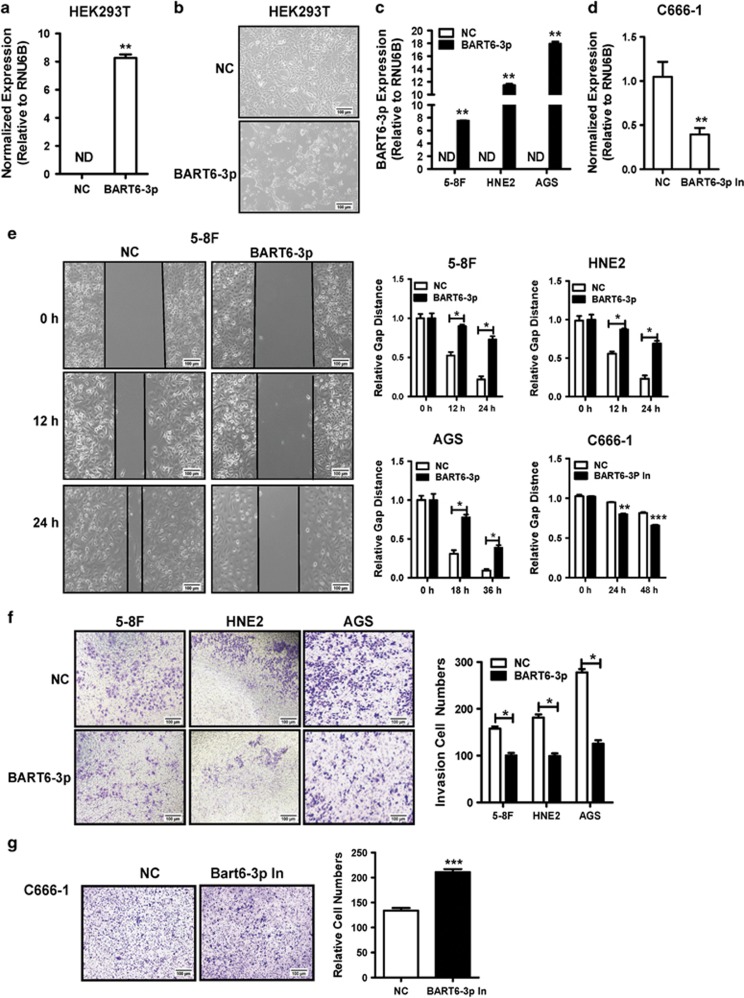
EBV-miR-BART6-3p mimics suppresses tumor cell migration and invasion *in vitro*. The bars indicate mean±S.D. (*n*=3). *P<0.05; **P<0.01; ***P<0.001. (**a**) Exogenous EBV-miR-BART6-3p expression was detected by real-time PCR after transfection of HEK293T cells with EBV-miR-BART6-3p mimics (BART6-3p) or negative control (NC). ND, not detectable. (**b**) Morphological alterations in HEK293T cells upon BART6-3p mimic transfection, as assessed by phase contrast microscopy ( × 100). (**c**) Expression of exogenous EBV-miR-BART6-3p was detected in HNE2, 5-8 F and AGS cells after transfection with EBV-miR-BART6-3p mimics (BART6-3p) or negative control (NC) in EBV-negative NPC cell lines (HNE2 and 5-8 F) or gastric cell line (AGS). (**d**) Expression of exogenous EBV-miR-BART6-3p was detected in C666-1 cells after transfection with EBV-miR-BART6-3p inhibitor (BART6-3p In) or negative control (NC) in EBV-positive cell line (C666-1). (**e**) EBV-miR-BART6-3p mimics inhibited 5-8 F, HNE2, and AGS cell migration. EBV-miR-BART6-3p inhibitors accelerated C666-1 cell migration. Cells were grown and transfected with EBV-miR-BART6-3p mimics, EBV-miR-BART6-3p inhibitors or a negative control and subjected to wound healing assays. The left panel shows the representative results of 5-8 F, and the right panel summarizes the relative width ratio of the wound healing gap from three independent experiments. (**f**) EBV-miR-BART6-3p mimics inhibited tumor cell invasion as measured by transwell matrigel penetration assay. 5-8 F, HNE2 and AGS cells were grown and transfected with EBV-miR-BART6-3p mimics or negative control for 36 h and were subjected to a matrigel invasion assay (left panel). The right graph summarizes the data from three independent experiments. (**g**) EBV-miR-BART6-3p inhibitors accelerated C666-1 cell invasion as measured by transwell matrigel penetration assay. C666-1 cells were grown and transfected with EBV-miR-BART6-3p inhibitors or negative control for 48 h and were subjected to a matrigel invasion assay (left panel). The right graph summarizes the data from three independent experiments

We previously established a comprehensive EBV-miRNA profiles in NPC and found that 40 EBV miRNAs from the BART transcript were highly expressed in NPC.^23^ To determine the biological functions of the BART cluster miRNAs, we transfected these miRNAs one by one into HEK293T cells. Interestingly, we found that overexpression of EBV-miR-BART6-3p in HEK293T cells (Figure 1a) dramatically altered HEK293T cell shape (Figure 1b). The negative control (NC) cells exhibited abundant pseudopods and filopodia, and the cells were elongated and spindle-shaped. Upon transfection with EBV-miR-BART6-3p mimics, the pseudopods and filopodia thickened and short in a portion of cells, while the filopodia virtually disappeared in some cells (Figure 1b). Because pseudopods and filopodia are essential for cell migration, we investigated in detail whether EBV-miR-BART6-3p affected cancer cell metastasis and invasion in this study. We firstly transfected EBV-miR-BART6-3p mimics into three EBV-negative cancer cell lines, 5-8 F and HNE2 (both NPC cell lines), and AGS (a gastric cancer cell line, as some gastric cancers are caused by EBV) (Figure 1c) to examine the effects of EBV-miR-BART6-3p on cell migration and invasion. On the other hand, EBV-miR-BART6-3p inhibitors were transfected into EBV-positive NPC cell line C666-1 (Figure 1d). Then we evaluated the effect of EBV-miR-BART6-3p mimics in three EBV-negative cancer cell lines or EBV-miR-BART6-3p inhibitors in EBV-positive C666-1 cell lines on cell migration by the cell scratch test (Figure 1e) and cell invasion by transwell assay (Figures 1f and g). Cell migration speed and the number of invasive cells significantly decreased in cancer cell lines after EBV-miR-BART6-3p mimic transfection, while EBV-miR-BART6-3p inhibitors promote cancer cell migration and invasion, suggesting that EBV-miR-BART6-3p inhibited the invasion and metastasis of EBV-associated epithelial cancer cells.

### EBV-miR-BART6-3p directly targeted and downregulated LOC553103

To explore the mechanism of EBV-miR-BART6-3p-mediated inhibition of cell migration and invasion, we attempted to identify its targets. Firstly, we did bioinformatics analysis to screen potential targets for EBV-miR-BART6-3p. There were 738 genes and were predicted as potential targets of EBV-miR-BART6-3p with low minimum free energy (MFE) (<−20.0) screened by the Reptar software (http://reptar.ekmd.huji.ac.il/). Then these potential targets were confirmed by the RNAhybrid software (http://bibiserv.techfak.uni-bielefeld.de/rnahybrid/) and *TUSC2* and *VANGL2* genes were selected for further study because of their lower MFE. Unfortunately the luciferase assay showed that the luciferase activity of *TUSC2* and *VANGL2* had no significant change upon EBV-miR-BART6-3p mimic transfection (Supplementary Figure S1A).Then we used the whole-genome microarray, which contains probes for known human protein coding genes (mRNAs) and long non-coding RNA genes (lncRNAs) to identify dysregulated genes by EBV-miR-BART6-3p in HEK293T cells. There were 1088 mRNAs (591 were downregulated and 497 were upregulated) and 2841 lncRNAs (1580 were downregulated and 1261 were upregulated) that were dysregulated by >1.5-fold and the false discovery ratio (FDR) was <0.05 in EBV-miR-BART6-3p mimics transfection compared with the NC transfection. Detailed information on dysregulated genes and their expression data is shown in Supplementary Table S1. *WTX* and *TPPP1* genes were downregulated by EBV-miR-BART6-3p and also were predicted as potential targets of EBV-miR-BART6-3p with low MFE. The luciferase assay showed that the luciferase activity of *WTX* and *TPPP1* was significantly reduced by EBV-miR-BART6-3p (Supplementary Figure S1B). So we chose real-time PCR and western blotting to examine *WTX* and *TPPP1* expression in 5-8 F, HNE2 and AGS cells after transfection with EBV-miR-BART6-3p mimics. The inhibition effect of EBV-miR-BART6-3p mimics on *WTX* and *TPPP1* was not consistent in 5-8 F, HNE2 and AGS cells (Supplementary Figure S1C), but TPPP1 expression was inhibited at the protein level but not at the mRNA level in all three cell lines (Supplementary Figure S1D). Moreover, *TPPP1* overexpression could not reverse EBV-miR-BART6-3p function in 5-8 F, HNE2 and AGS cells (data not shown). These results suggested that *WTX* and *TPPP1* genes were not a bona fide target of EBV-miR-BART6-3p.

**Figure 2:**
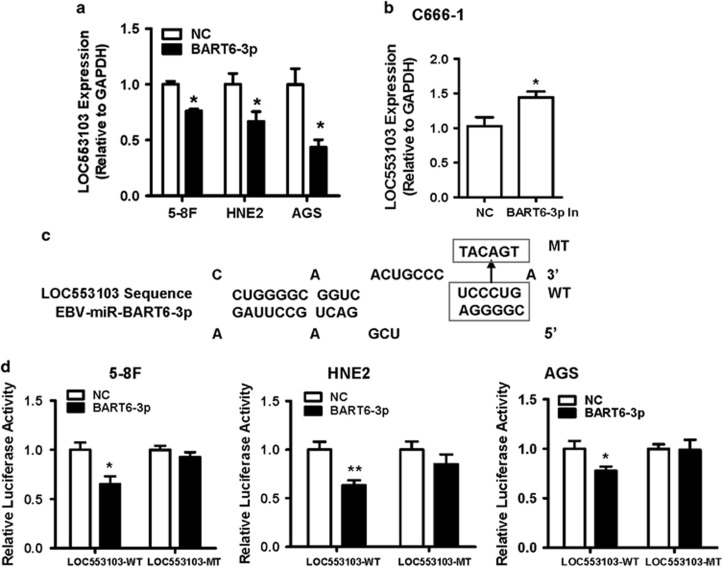
LOC553103 is a direct EBV-miR-BART6-3p target. The bars indicate mean±S.D. (*n*=3). *P<0.05; **P<0.01; ***P<0.001. (**a**) *LOC553103* expression was inhibited by EBV-miR-BART6-3p mimics in 5-8 F, HNE2 and AGS cells. (**b**) *LOC553103* expression was induced by EBV-miR-BART6-3p inhibitors in C666-1 cells. (**c**) The location of the possible seed-matched sites for EBV-miR-BART6-3p on *LOC553103* sequence and the sites changed to produce mutated forms of LOC553103. The EBV-miR-BART6-3p seed region had a complementary binding site in the *LOC553103* sequence. For the mutated (Mut) form, the potential binding nucleotides were replaced to disrupt the complementarities. (**d**) 5-8 F, HNE2 and AGS cells were transiently co-transfected with EBV-miR-BART6-3p (BART6-3p) or negative control (NC) as well as pRL-TK and luciferase reporters containing either wild type (LOC553103*-*WT) or mutated (LOC553103-mut) EBV-miR-BART6-3p-binding site. The cells were analyzed for luciferase activity after 48 h. The experiments were repeated three times, and the error bars denote the mean. EBV-miR-BART6-3p mimics attenuated LOC553103-WT luciferase activity compared with the LOC553103*-*mutant

Therefore, we focused on those lncRNAs that were downregulated by EBV-miR-BART6-3p mimics in the microarray data. Three lncRNAs, including *LINC00461*, *SRGAP2-AS1* and *LOC553103*, were significantly downregulated by EBV-miR-BART6-3p mimics and also contained EBV-miR-BART6-3p-binding sites predicted by bioinformatics analysis. We firstly validated the expression of these lncRNAs in 5-8 F, HNE2 and AGS after EBV-miR-BART6-3p mimics transfection by real-time PCR. Among them, *LINC00461* expression was not significantly decreased in NPC and GC cells, while *SRGAP2-AS1* expression increased after EBV-miR-BART6-3p mimics transfection, which was inconsistent with our microarray data in HEK293T cells (Supplementary Figure S2). In contrast, *LOC553103* expression was significantly repressed by EBV-miR-BART6-3p in all three cancer cell lines tested (Figure 2a). And EBV-miR-BART6-3p inhibitors could upregulate the expression of *LOC553103* in EBV-positive C666-1 cells (Figure 2b). These results suggested *LOC553103* was a potential target of EBV-miR-BART6-3p. Using online miRNA target prediction software and databases (RNAHybrid and Reptar), we identified a target site in *LOC553103* with the MFE upon EBV-miR-BART6-3p binding (Figure 2c). To confirm whether the predicted EBV-miR-BART6-3p-binding site within the *LOC553103* sequence was responsible for downregulating *LOC553103* expression, we mutated the EBV-miR-BART6-3p-binding site of *LOC553013* sequence and performed luciferase assays. The luciferase reporter vector and EBV-miR-BART6-3p mimics or scramble NC were co-transfected into 5-8 F, HNE2 and AGS cells. The results showed that the luciferase activity of the LOC553103-WT vector was significantly reduced by addition of the EBV-miR-BART6-3p mimics compared with NC cells. However, EBV-miR-BART6-3p-mediated repression of luciferase activity was abolished when cells were transfected with the vector containing the mutant-binding site (LOC553103-MT) (Figure 2d). These results suggested that direct binding of EBV-miR-BART6-3p to lncRNA *LOC553103* led to *LOC553103* expression decreased.

### LOC553103 overexpression promoted cancer cell metastasis and invasion

**Figure 3:**
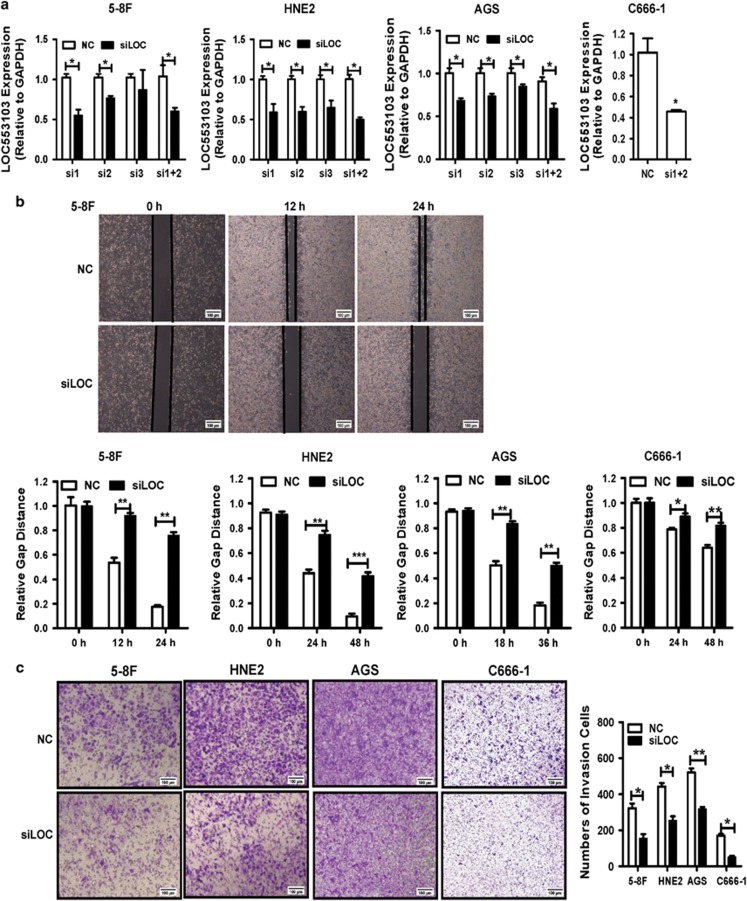
LOC553103-siRNA knockdown inhibits cancer cell migration and invasion *in vitro*. The graph summarizes the data from three independent experiments. The bars indicate mean±S.D. (*n*=3). *P<0.05; **P<0.01; ***P<0.001. (**a**) The efficiency of *LOC553103* knockdown was measured by examining *LOC553103* expression in 5-8 F, HNE2 and AGS cells 48 h after transfection with the *siLOC553103* or the scrambled control were measured by real-time PCR. *GAPDH* was used as an internal control for real-time PCR. All three siRNA sequences (si1, si2 and si3) decreased *LOC553103* expression in 5-8 F, HNE2 and AGS cells, but siRNA1 and siRNA2 had better effect than siRNA3, so the mixture of siRNA1 and siRNA2 was used for *LOC553103* knockdown in subsequent experiments. The efficiency of *LOC553103* knockdown was also measured by examining LOC553103 expression in C666-1 cells transfected with the mixture of siRNA1 and siRNA2. (**b**) *LOC553103* knockdown inhibited 5-8 F, HNE2, AGS and C666-1 cell migration. Cells were grown and transfected with *LOC553103* siRNA (siLOC) or scrambled negative control (NC) and then subjected to the wound healing assay. The upper panel shows the representative results of 5-8 F, and the lower panel summarizes the relative width ratio of the wound healing gap from three independent experiments. (**c**) *LOC553103* knockdown inhibited tumor cell invasion as measured by transwell matrigel penetration assay. 5-8 F, HNE2, AGS and C666-1 cells were grown and transfected with *LOC553103* siRNAs (siLOC) or scrambled negative control (NC) and then subjected to a matrigel invasion assay

To further confirm whether EBV-miR-BART6-3p exerted its biological function directly through *LOC553103* downregulation and evaluate the effect of *LOC553103* on cancer cell metastasis and invasion, we first examined whether siRNA knockdown of lncRNA *LOC553103* phenocopied EBV-miR-BART6-3p expression, which inhibited cancer cell metastasis and invasion. We synthesized three siRNA sequences targeting *LOC553103*, and all three siRNAs significantly knocked down *LOC553103* expression compared with the scrambled negative control (NC) in 5-8 F, HNE2 and AGS cells (Figure 3a). The mixture of siRNA1 and siRNA2 was used for all subsequent experiments because of their better knockdown effects. The mixture of siRNA1 and siRNA2 could also significantly knock down *LOC553103* expression in EBV-positive C666-1 cells (Figure 3a). Cells transfected with siRNAs against lncRNA *LOC553103* were plated into six-well plates and performed *in vitro* migration assays. The data showed that knockdown of *LOC553103* significantly inhibited cell migration in EBV-positive or -negative cancer cells (Figure 3b), and *LOC553103* knockdown significantly inhibited cell invasion (Figure 3c).

**Figure 4:**
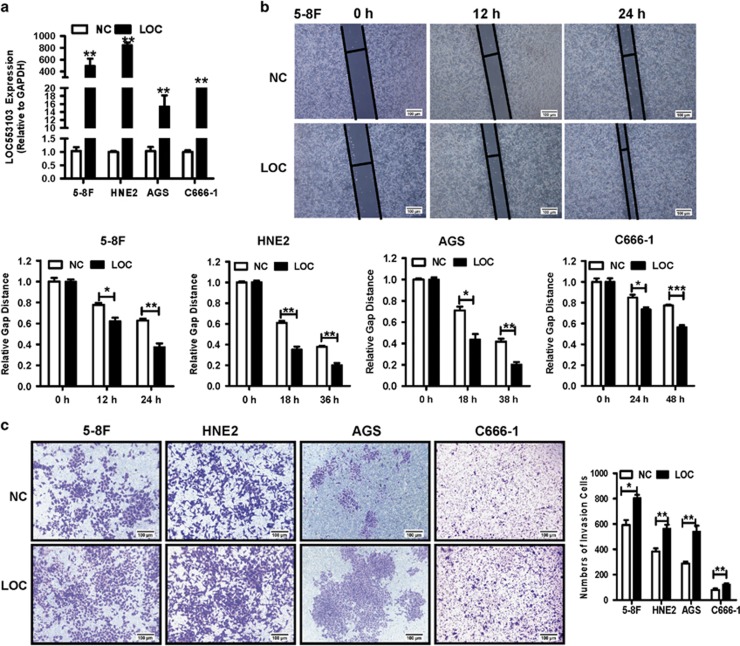
LOC553103 overexpression promotes cancer cell migration and invasion *in vitro*. The graph summarizes the data from three independent experiments. The bars indicate mean±S.D. (*n*=3). *P<0.05; **P<0.01; ***P<0.001. (**a**) *LOC553103* expression levels in 5-8 F, HNE2, AGS and C666-1 cells were measured by real-time PCR 48 h after transfection with the *LOC553103* overexpression vector (LOC) or negative control empty vector (NC). *GAPDH* was used as an internal control. (**b**) LOC553103 induced 5-8 F, HNE2, AGS and C666-1 cell migration. Cells transfected with *LOC553103* overexpression vector (LOC) or negative control empty vector (NC) were subjected to the wound healing assay. The upper panel shows representative results of 5-8 F, and the lower panel summarizes the relative width ratio of the wound healing gap from three independent experiments. (**c**) LOC553103 promoted tumor cell invasion as measured by transwell matrigel penetration assay. 5-8 F, HNE2, AGS and C666-1 cells were transfected with *LOC553103* overexpression vector (LOC) or the empty vector (NC) and then subjected to a matrigel invasion assay (left panel)

On the other hand, the *LOC553103* overexpression vector was constructed and transfected into 5-8 F, HNE2, AGS and C666-1 cells to confirm its function on cell migration and invasion. Real-time PCR confirmed the overexpression effect firstly (Figure 4a). Would healing and transwell invasion assays revealed that *LOC553103* overexpression promoted cell migration (Figure 4b) and invasion (Figure 4c), which reversed the EBV-miR-BART6-3p mimic phenotype and the LOC553013 knockdown phenotype. These results suggested that EBV-miR-BART6-3p inhibited cancer cell metastasis and invasion by targeting and downregulating LOC553103.

### EBV-miR-BART6-3p overexpression and LOC553103 knockdown inhibited metastasis in nude mice

**Figure 5:**
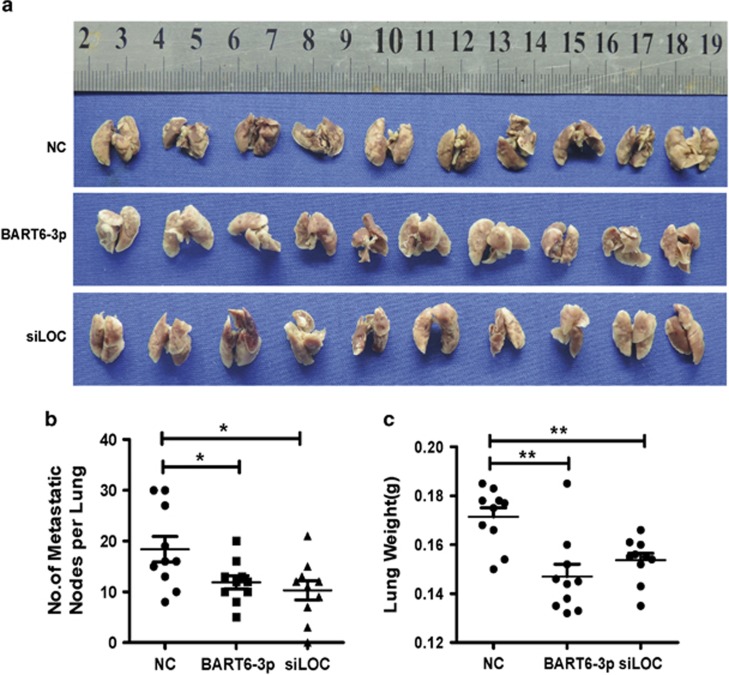
EBV-miR-BART6-3p overexpression or LOC553103 knockdown inhibits metastasis in nude mice. (**a**) A total 30 BALB/c nude mice aged 4 weeks were divided into three groups (10 mice per group), and each mouse was injected with 1 × 10^6^ 5-8 F cells transfected with EBV-miR-BART6-3p mimics (BART6-3p), *LOC553103* siRNAs (siLOC) or scrambled control sequence (NC) into the tail vein. After 2 months, the mice were killed, and their lungs were removed for assessment of the metastasized tumor nodules. (**b**) Numbers of metastasized tumor nodules in the lung per mouse were counted. (**c**) Mouse lung weights were scaled and reflected the decreased metastasized tumor foci size

To confirm the effects of EBV-miR-BART6-3p overexpression or *LOC553103* knockdown *in vivo*, 5-8 F cells transfected with EBV-miR-BART6-3p, *LOC553103* siRNA or scrambled control siRNA as a negative control (NC) were inoculated into the tail veins of nude mice and assessed the number of metastasized tumor nodules in the lung. Both EBV-miR-BART6-3p and *LOC553103* siRNA significantly reduced the size and number of metastasized tumor foci (Figure 5a). We detected tumor nodules on the lung surface in 100% (10/10) of mice inoculated with 5-8 F control-siRNA cells, with an average of 18.4±7.94 nodules per mouse. There were also 100% (10/10) of mice inoculated with 5-8 F EBV-miR-BART6-3p and 90% (9/10) mice inoculated with LOC553103-siRNA cells. However, significantly fewer nodules, with an average of 11.9±4.14 nodules per mouse in the EBV-miR-BART6-3p group and 10.3±5.98 nodules in the LOC553103-siRNA group, were detected (Figure 5b). The mouse lungs were also weighted and reflected the decreased metastasized tumor foci size in mice inoculated with the EBV-miR-BART6-3p or LOC553103-siRNA groups (Figure 5c).

### EBV-miR-BART6-3p and LOC553103 regulated EMT and metastasis-related genes expression

**Figure 6:**
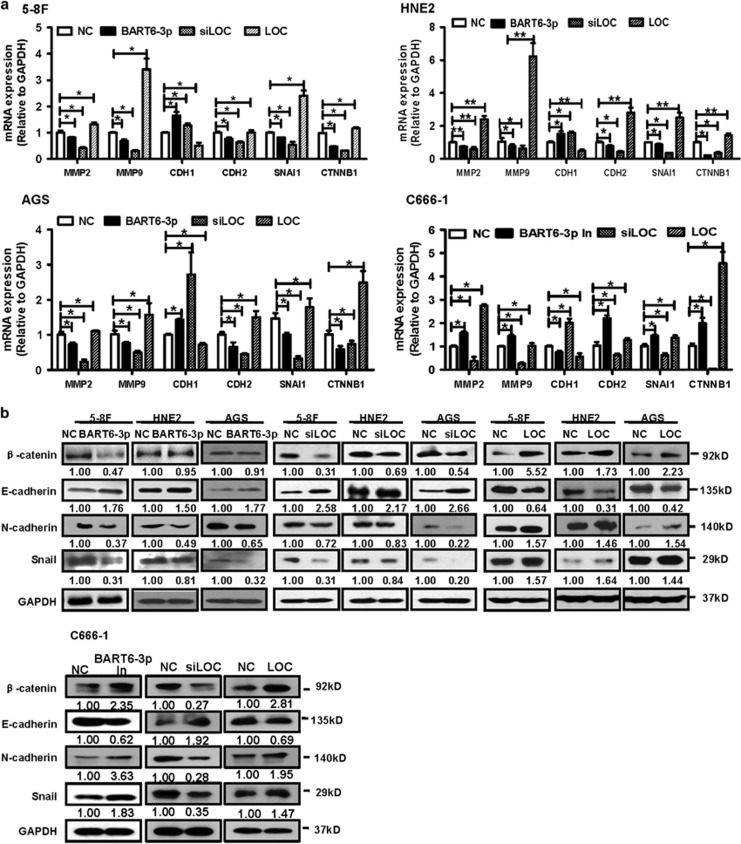
EBV-miR-BART6-3p and LOC55310 regulate EMT and metastasis-related gene expression. (**a**) 5-8 F, HNE2, AGS or C666-1 cells were transfected with EBV-miR-BART6-3p mimics (BART6-3p) or EBV-miR-BART6-3p inhibitors (BART6-3p In), *LOC553103* siRNAs (siLOC) or *LOC553103* overexpression vector. Forty-eight hours after transfection, cells were harvested and mRNA expression levels of metastasis-related genes MMP2, MMP9, EMT-related genes CDH1 (encoding E-cadherin protein), CDH2 (encoding N-cadherin) SNAL1 and CTTNB1 (encoding *β*-catenin) were measured by real-time PCR. (**b**) Important EMT markers *β*-catenin, E-cadherin, N-cadherin and Snail protein expression were measured by western blot 48 h after transfection with EBV-miR-BART6-3p mimics (BART6-3p) or EBV-miR-BART6-3p inhibitors (BART6-3p In), *LOC553103* siRNAs (siLOC) or *LOC553103* overexpression vector in 5-8 F, HNE2, AGS or C666-1 cells

In order to examine the molecular mechanism in detail of EBV-miR-BART6-3p, we examined whether EBV-miR-BART6-3p affected the expression of metastasis-associated proteins, especially EMT markers. Real-time PCR showed that exogenous EBV-miR-BART6-3p mimics increased expression of the epithelial marker E-cadherin (CDH1) and decreased the mesenchymal marker N-cadherin (CDH2), and the transcription factor *β*-catenin and Snail (encoded by the SNAL1 gene) in EBV-negative tumor cell lines (5-8 F, HNE2 and AGS) at the mRNA level (Figure 6a). Furthermore, EBV-miR-BART6-3p mimics inhibited metastasis and invasion-related genes, including *MMP2* and *MMP9* in three EBV-negative cancer cell lines (Figure 6a). Western blotting also showed the increased E-cadherin protein expression and decreased CDH2, Snail and *β*-catenin protein expression with EBV-miR-BART6-3p mimics treatment (Figure 6b). On the other hand, EBV-miR-BART6-3p inhibitors had the opposite effect on these metastasis markers and invasion-related genes in EBV-positive NPC C666-1 cells (Figure 6). *LOC553103* knockdown phenocopied the effect of EBV-miR-BART6-3p mimics on the EMT and metastasis marker expression, while *LOC553103* overexpression had the opposite effects in four cell lines (5-8 F, HNE2, AGS and C666-1 (Figure 6b).

### EBV-miR-BART6-3p and LOC553103 siRNAs induced loss of stress fiber integrity in cancer cells

**Figure 7:**
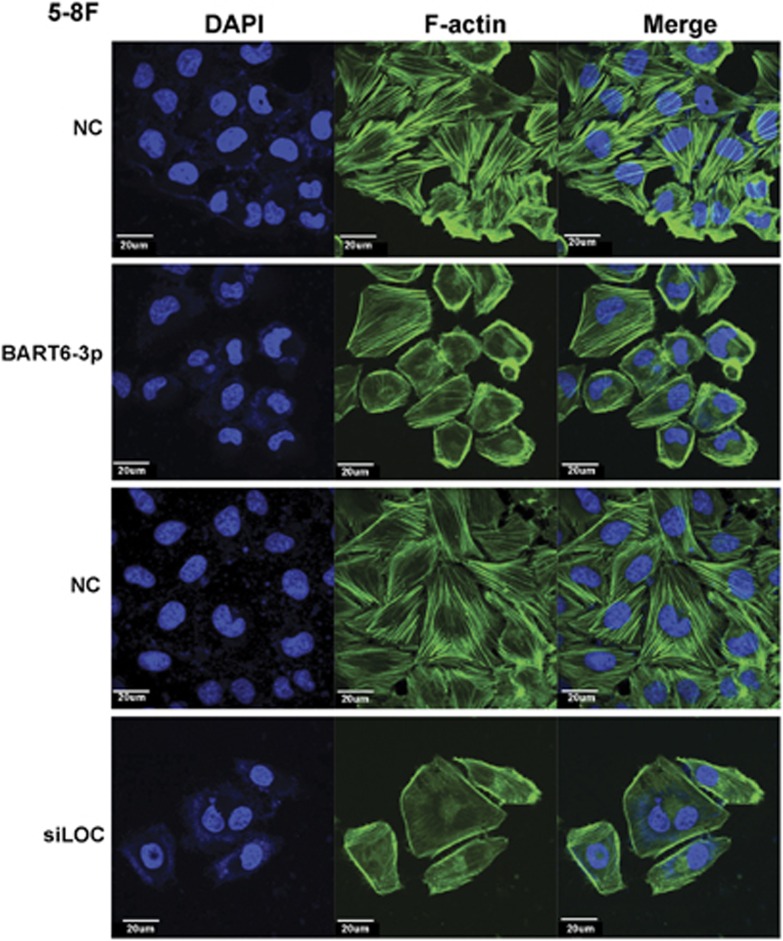
Transfection of EBV-miR-BART6-3p or LOC55310 siRNAs destroys stress fiber integrity in 5-8 F cells. 5-8 F cells were transfected with EBV-miR-BART6-3p (BART6-3p) mimics, *LOC553103* siRNAs (siLOC) or scrambled control (NC), and 48 h after transfection, cells were fixed and stained for F-actin by phalloidin (green), and 4′,6-diamidino-2-phenylindole was used to stain nuclei (blue). A clear deficiency in stress fiber formation was observed in BART6-3p mimic and siLOC553103-transfected 5-8 F cells. Images were acquired at × 400. Scale bar=20 *μ*m

Cell actin cytoskeleton is an essential component of pseudopod and filopodia formation and tightly associated with cancer metastasis. We investigated whether EBV-miR-BART6-3p or *LOC553103* regulated actin filament integrity. Immunofluorescence demonstrated that unlike scrambled control-treated 5-8 F cells, phalloidin did not label stress fibers after EBV-miR-BART6-3p mimic or *siLOC553103* transfection, suggesting that EBV-miR-BART6-3p affected 5-8 F cell stress fiber integrity by repressing *LOC553103* (Figure 7).

## Discussion

EBV produces several viral oncoproteins, including six EBV-encoded nuclear antigen proteins (EBNA-1, EBNA-2, EBNA-3A, 3B, 3C and EBNA-LP) and three latent membrane proteins (LMP1, LMP2A, and LMP2B) and 44 miRNAs.^23^ The BART cluster miRNAs of EBV were high expressed in NPC and ^GC23, 24, 25^ and most of BART cluster miRNAs such as BART10-3p, BART11, BART1-3p, BART7-3p and BART9 acted as oncogenes and promoted nasopharyngeal epithelium malignant transformation.^19, 26, 27, 28, 29, 30^ However, this study found that EBV-miR-BART6-3p, a microRNA encoded by oncogenic EBV, acted as a tumor suppressor by targeting and downregulating a novel lncRNA LOC553103. In fact, other than encoding oncogenes and promoting nasopharyngeal epithelium malignant transformation, some genes encoded by EBV could also inhibit the phenotype of NPC cells such as Rta and Zta, two transcription factors expressed by EBV during the immediate-early stage of the lytic cycle, could inhibit cell proliferation and result in cell cycle arrest through induction of cyclin-dependent kinase inhibitors.^9, 31, 32, 33, 34, 35, 36^ Some studies also reported that another EBV miRNA, EBV-miR-BART15-3p, exerted their suppressive function and ‘anti-cancer' activities through targeting cellular genes mainly for preventing apoptosis and escaping the host immune system.^37^ In this sense, not only all the genes encoded by EBV have oncogenes' function but also some have tumor suppressor genes' function. The detailed role of EBV-miR-BART6-3p as a tumor suppressor gene in EBV-associated cancers needs our further more research.

EBV infection has been linked to several types of tumors.^38^ In this study, we found that EBV-miR-BART6-3p inhibited migration and invasion of NPC and GC cells. However, BART6-3p may exert growth-inducing properties and affect the immune response in EBV-positive Burkitt lymphoma through affecting the function of important signal transducers as NF*κ*B and Akt/PI3K or by downregulating PTEN to remove the inhibitory brake on cell proliferation.^21^ Thus our data confirmed that different pathogenetic mechanisms may exist in EBV-associated epithelial malignant transformation and Burkitt's lymphomagenesis. EBV-miR-BART6-5p, another EBV-encoded miRNA, was reported to silence Dicer through multiple target sites located in the 3′-UTR of Dicer mRNA. It also suppressed the EBNA-2 viral oncogentargeting for transition from immunologically less responsive type I and type II latency to the more immunoreactive type III latency as well as Zta and Rta viral proteins essential for lytic replication, revealing the regulatory function of miR-BART6 in EBV infection and latency.^20^ The function of miR-BART6-3p in this study suggested that miR-BART6-3p might also have functions to regulate EBV infection and latency.

In this study, the microarray profile and bioinformatics prediction were combined to identify EBV-miR-BART6-3p targets. Through a large number of mRNAs and lncRNAs were screened and tested and LOC553103, a novel lncRNA, was confirmed as a target of EBV-miR-BART6-3p. Dysregulation of non-coding RNAs, including lncRNAs and miRNAs, has been frequently implicated in cancers. A growing body of evidence has demonstrated the interplay between miRNAs and lncRNAs.^39^ Tsang *et al.*^40^ demonstrated that *HOTTIP* was a novel oncogenic lncRNA, which was negatively regulated by miR-125b. miR-21 has been shown to negatively regulate lncRNA *GAS5* in breast cancer.^41^ To our knowledge, our study is the first report of a virus miRNA that suppresses a host human lncRNA to affect cancer cell metastasis. However, it remains unclear what the downstream molecular mechanisms and signaling pathways of *LOC553103* are. In other words, it is indeterminate how *LOC553103* regulates signaling pathways to promote EMT and enhances cancer cell invasion and migration. The recognition of cross-talk between viral miRNAs and lncRNAs is of particular importance because it provides theoretical and practical relevance for the influence of EBV infection on carcinogenesis. These results will be of great use in the treatment of NPC and other EBV-related cancers.

**Figure 8:**
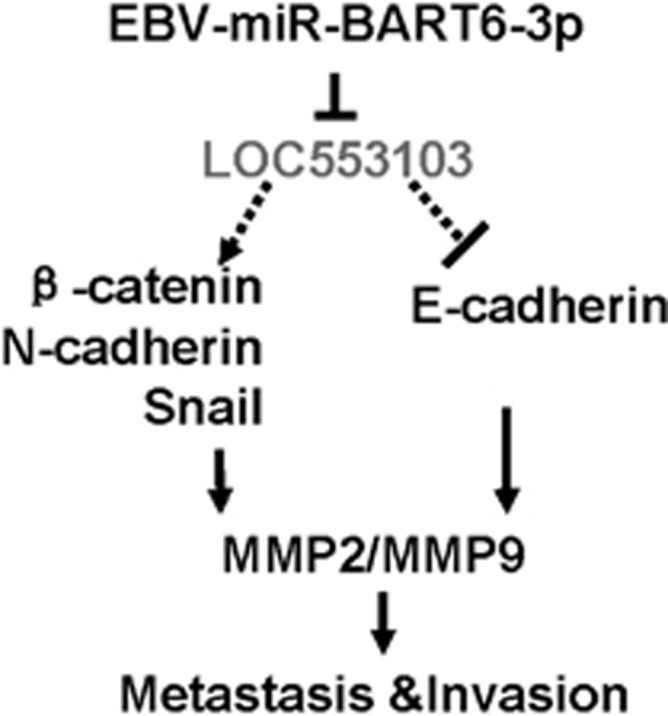
A schematic model of EBV-miR-BART6-3p functioning on cancer invasion and migration and stress fiber integrity through inhibition of its target *LOC553103*

In conclusion, our study revealed that an EBV miRNA, EBV-miR-BART6-3p, could inhibit invasion and migration of EBV-associated cancer cells and change stress fiber integrity, through inhibition of its target *LOC553103* expression, leading to the regulation of many EMT-related molecules, such as upregulated expression of E-cadherin, as well as downregulated *β*-catenin, Snail, and N-cadherin and metastasis markers and invasion-related genes, including *MMP2* and *MMP9* (Figure 8). Our study provided for the first time evidences that a new mechanism of EBV infection in EBV-positive and EBV-negative cancer cells and that EBV-miR-BART6-3p significantly affects cancer cell molecular phenotype by opening new scenarios.

## Materials and Methods

### Cell lines, transfection, plasmids and chemicals

EBV-negative nasopharyngeal carcinoma (NPC) cell lines (5-8 F and HNE2), EBV-positive NPC cell line (C666-1) and EBV-negative gastric cancer (GC) cell line AGS were grown in RPMI 1640 (HyClone, Logan, UT, USA) supplemented with 10% fetal bovine serum, 100 U/ml penicillin and 100 *μ*g/ml streptomycin (Gibco, Grand Island, NY, USA). HEK293T cells were grown in Dulbecco's modified Eagle's medium. Cultures were maintained at 37 °C in a humidified environment with 5% CO_2_. EBV miRNAs mimics and negative siRNA control were purchased from Qiagen Company (Valencia, CA, USA). LOC533103 siRNA was synthesized by Ruibo Company (Guangzhou, China). Hiperfect transfection reagent (Qiagen) was used for transfection of miRNA mimics or oligonucleotides for RNA interference (RNAi). The cDNA encoding lncRNA *LOC553103* was PCR-amplified and subcloned into the *Bam*H1 and EcoRI sites of the pcDNA3.1 vector (Invitrogen, Breda, The Netherlands), named pcDNA3.1-*LOC553103* using Magic Cloning Biological Reagent (Light of Life Biotechnology Co., Ltd, Changsha, China). Plasmid transfections were performed using Lipofectamine 3000 (Invitrogen) according to the manufacturer's instructions.

### Quantitative real-time PCR

Total RNA was extracted and purified using TRIzol (Invitrogen) according to the manufacturer's instructions. For miRNA expression analysis, real-time PCR was performed using the miScript system (Qiagen) and Qiagen miRNA primer assays (Qiagen) using the miScript SYBR Green PCR Kit (Qiagen) according to the manufacturer's protocol. Human U6 served as the internal control. For miRNA expression analysis, real-time PCR was performed using SYBR Premix Ex Taq (Takara, Dalian, China) on a real-time PCR system (Biorad, Hercules, CA, USA). Primer sequences are listed in Supplementary Table S2. *GAPDH* was used as the reference gene for normalization of all gene expression results. The average of three independent analyses for each gene was calculated. The fold changes were calculated through relative quantification (2^−ΔΔCt^). All reactions were run in triplicate and repeated in three independent experiments.

### Bioinformatics analysis and luciferase assay

We did bioinformatics analysis to predict potential targets of EBV-miR-BART6-3p using two publicly available algorithms: Reptar (http://reptar.ekmd.huji.ac.il/) and RNAhybrid (http://bibiserv.techfak.uni-bielefeld.de/rnahybrid/) software. The fragments from *LOC553103* containing wild-type EBV-miR-BART6-3p putative target seed sites and their mutants (Figure 2c) were synthesized (Invitrogen) and annealed into a mir-Reporter luciferase vector containing firefly luciferase (Promega, Madison, WI, USA). The mutants were designed and synthesized, in which six bases in the binding site were substituted.^23^ These constructs were co-transfected into 5-8 F, HNE2 or AGS cells in 24-well plates together with 20 nM EBV-miR-BART6-3p mimics or negative control siRNA. Luciferase activity was measured 48 h after transfection using the dual-luciferase reporter assay system (Promega). Firefly luciferase activity was used to normalize Renilla luciferase activity for each transfected well. These experiments were repeated three times, and the *P*-value was calculated by *t*-test analysis.

### Gene expression microarray

Microarray analysis was performed by the OE Human lncRNA Microarray V2.0 (Oebiotech, Shanghai, China) that contains approximately 46 506 lncRNAs and 30  656 mRNAs (Oebiotech, http://www.oebiotech.com/). LncRNA information came from NCBI, Ensembl, LNCipedia, NonCodeV4, lncRNAdb, Broad institute (Human Body Map lincRNAs) and GeneCode database. For chip hybridization, total RNA samples isolated from HEK293T cells transfected with EBV-miR-BART6-3p mimics or negative siRNA control using TRIzol (Invitrogen) and further purified with an RNeasy Mini Kit (Qiagen) following the manufacturer's instructions. Gene expression profiling was carried out as described previously.^42^ Briefly, cRNA was synthesized by T7 RNA polymerase and labeled with Cy3-CTP, then hybridized to the microarray. After hybridization and washing, processed slides were scanned with an Agilent Microarray Scanner (Agilent Technologies, Santa Clara, CA, USA), and the acquired array images were analyzed using Agilent Feature Extraction Software (Agilent Technologies), which performs background subtractions. Quantity normalization and subsequent data processing were performed using the GeneSpring GX v.11.0 software package (Agilent Technologies). Significant Analysis of Microarray (SAM) software^43^ was used to analyze dysreuglated mRNA and lncRNAs. Threshold fold changes of >1.5 and the FDR was <0.05 were used to screen for dysregulated probes. The lncRNA and miRNA expression data were deposited in the Gene Expression Omnibus database (GEO, www.ncbi.nlm.nih.gov/geo/) under accession numbers GSE81528.

### Migration and invasion assays

*In vitro* migration and invasion assays were performed using transwell chambers as described previously with minor modifications.^44^ In brief, cells were serum-starved after 24 h of transfection, and detached and resuspended in serum-free medium for 24 h again. A 100-*μ*l cell suspension was added to the upper chamber (8.0 mM pore size; Corning, NY, USA), and medium supplemented with 20% FCS was added to the bottom chamber. Cells on the upper surface of filters were removed after 36 h, and those on the undersurface were stained with 5% crystal violet. Images were captured from each membrane, and the number of invasive cells was counted under a microscope.

For wound healing assays, cells were seeded into 6 cm culture dishes and grown to a near-confluent monolayer. A 10 *μ*l pipet tip was used to scratch a line across the middle of each dish, and cellular debris was removed by washing with phosphate-buffered saline (PBS). The cultures were incubated at 37 °C and photographed. The number of cells that migrated into the cell-free zone was scored and evaluated. Each sample was assayed in triplicate, and a minimum of three independent experiments were performed.

### Animal experiments

Four-week-old female BALB/c nude mice were purchased from the Laboratory Animal Services Centre of Central South University. Animal handling and experimental procedures were approved by the Animal Experimental Ethics Committee of Central South University. To determine the lung metastatic potential of cancer cells *in vivo*, we injected 1 × 10^6^ 5-8 F/BART6-3p, 5-8 F/*siLOC553103* or 5-8 F/NC cells into nude mice (*n*=10/group) through the tail vein. The mice were all killed 2 months later, at which time individual organs were removed.

### Western blotting

Western blotting was performed as described previously.^26^ Samples were separated by electrophoresis on 10–12% sodium dodecyl sulfate polyacrylamide gels, and the separated proteins were transferred to a polyvinylidene fluoride membrane (Millipore, Billerica, MA, USA). To assess protein expression, blots were incubated with primary antibodies at 4 °C overnight. Primary antibodies, including *β*-catenin, E-cadherin, Snail and GAPDH (Cell Signaling Technology, Danvers, MA, USA) were used, and immunoreactivity was visualized by the ECL western blotting detection system (GE Healthcare, Amersham, UK). Densitometric analysis of immunodetected bands was performed using Image Analysis software (Biorad).

### Immunofluorescence

Cells were fixed in 4% paraformaldehyde for 20 min, washed three times with PBS and blocked with PBS containing 7% fetal bovine serum for 30 min. Cells were incubated with Alexa Fluor 488 phalloidin (Molecular Probes, Eugene, OR, USA) in PBS for 1 h, washed three times with PBS, incubated with 4′,6-diamidino-2-phenylindole for 10 min at room temperature. Immunofluorescence images were collected using a confocal fluorescence microscope (UltraView Vox; PerkinElmer, Waltham, MA, USA).

### Statistical analysis

Data were analyzed using Student's *t*-test. Cell proliferation during the experimental periods was analyzed by two-way repeated-measure analysis of variance (ANOVA). Curve fitting analyses were performed using GraphPad Prism Software (GraphPad Software, San Diego, CA, USA). *P*<0.05 was considered statistically significant. All results were expressed as the mean±standard deviation (S.D.).

## Abbreviations

EBV: Epstein-Barr virus
lncRNA: long non-coding RNA
EMT: epithelial–mesenchymal transition
NPC: nasopharyngeal carcinoma
GC: gastric cancer
Rta: transcription activator
Zta: BZLF1 transcription activator
miRNAs: microRNAs
BART: the *Bam*HI A rightward transcript
NC: negative control
CDH1: E-cadherin
CDH2: N-cadherin
GEO: Gene Expression Omnibus database
WTX: APC membrane recruitment protein 1
TPPP: tubulin polymerization promoting protein
